# Wisdom of crowds and collective decision-making in a survival situation with complex information integration

**DOI:** 10.1186/s41235-020-00248-z

**Published:** 2020-10-15

**Authors:** Daisuke Hamada, Masataka Nakayama, Jun Saiki

**Affiliations:** 1 Graduate School of Human and Environmental Studies, Kyoto University, Yoshida, Nihonmatsu-cho, Sakyo-ku, Kyoto, 606-8501 Japan; 2 Kokoro Research Center, Kyoto University, Kyoto, Japan

**Keywords:** Group decision-making, Collective decision-making, Wisdom of crowds, Rank order, Subjective confidence

## Abstract

**Background:**

The wisdom of crowds and collective decision-making are important tools for integrating information between individuals, which can exceed the capacity of individual judgments. They are based on different forms of information integration. The wisdom of crowds refers to the aggregation of many independent judgments without deliberation and consensus, while collective decision-making is aggregation with deliberation and consensus. Recent research has shown that collective decision-making outperforms the wisdom of crowds. Additionally, many studies have shown that metacognitive knowledge of subjective confidence is useful for improving aggregation performance. However, because most of these studies have employed relatively simple problems; for example, involving general knowledge and estimating values and quantities of objects, it remains unclear whether their findings can be generalized to real-life situations involving complex information integration. This study explores the performance and process of the wisdom of crowds and collective decision-making by applying the wisdom of crowds with weighted confidence to a survival situation task commonly used in studies of collective decision-making.

**Results:**

The wisdom of crowds and collective decision-making outperformed individual judgment. However, collective decision-making did not outperform the wisdom of crowds. Contrary to previous studies, weighted confidence showed no advantage from comparison between confidence-weighted and non-weighted aggregations; a simulation analysis varying in group size and sensitivity of confidence weighting revealed interaction between group size and sensitivity of confidence weighting. This reveals that it is because of small group size and not the peculiarity of the survival task that results in no advantage of weighted confidence.

**Conclusions:**

The study’s findings suggest that the wisdom of crowds could be applicable to complex problem-solving tasks, and interaction between group size and sensitivity of confidence weighting is important for confidence-weighted aggregation effects.

## Significance Statement

The growth and prevalence of the Internet has resulted in an unprecedented system for gathering a large number of individual opinions. This system allows us to aggregate independent information and communicate face-to-face in online chat rooms. Correctly understanding and utilizing the wisdom of crowds, which aggregates information without consensus, and collective decision-making, which aggregates information with consensus, are urgent modern tasks to improve problem-solving efficiency, both in tasks with correct answers in open-ended tasks dependent on expert knowledge. Unlike most previous studies, which have addressed relatively simple problems, this study investigates the performance and process of the wisdom of crowds through a survival situation task involving complex information integration, and additionally compares with weighted subjective confidence and collective decision-making. The findings demonstrate the effective performance of the wisdom of crowds and collective decision-making and an effect of weighted confidence in interaction between group size and sensitivity of confidence weighting. This suggests that the wisdom of crowds can be applied and generalized to complex real-life situations. Weighted confidence based on large group size is compatible with a system that can collect a large number of opinions. Thus, this study expands the potential application of the wisdom of crowds to real-life problems involving complex information integration.

## Background

Decision-making in everyday life requires the integration of complex information. Decision-makers must integrate semantic knowledge under a current goal, understand the situation, qualitatively evaluate different behavior options, and select the optimal choice. Furthermore, this task is important not only for individuals but also for groups. Every human society relies on groups to make important decisions, as groups have more problem-solving resources than any individual member (Kerr and Tindale, 2004). Indeed, many tasks can be achieved only by a group effort and are beyond the capacity of a single individual or even many individuals working separately. For example, groups of business executives and politicians make decisions to maximize corporate and national interests in accordance with their specific goals. Examples can also be expanded to the global scale, such as policymaking between countries. This study investigates the performance and process of information integration with and without consensus in a situation requiring complex information integration.

How can information integration between individuals compensate for, and exceed, the limits of information integration within an individual? Previous studies have addressed two main aspects of information integration: the wisdom of crowds and collective decision-making. The wisdom of crowds is a phenomenon in which aggregation of many independent estimates without consensus often perform as well as, or better than, the majority of the individual decisions themselves (Surowiecki, 2004). Collective decision-making is a process whereby the members of a group decide on a course of action based on consensus (Montes de Oca et al., 2011). It has the potential to exceed the capacity of individual decision-making or simple aggregation of individual actions or competencies through social interactions that facilitate the emergence of collective choices (Krause, Ruxton and Krause, 2010). The two types of information integration have often been studied separately; additionally, recent research has begun to compare and combine them to explore the best information integration performance. Lee and Shi (2010) considered people’s ability to estimate the price of familiar household items and showed that the average estimates of three random, independent individuals were more accurate than collective estimates made by three-person groups. Navajas, Niella, Garbulsky, Bahrami and Sigman (2018) presented five-person groups with eight general knowledge questions involving estimation of uncertain numbers (e.g., the height of the Eiffel Tower; the number of elevators in New York’s Empire State Building) and showed that consensus estimates were more accurate than aggregation of individual estimates, and that averaging consensus decisions was substantially more accurate than aggregating initial independent opinions. Thus, comparing and combining collective decision-making with the wisdom of crowds is a useful method to determine effective aggregation algorithms.

Another important method involves metaknowledge of subjective confidence, which has often been applied to information integration in both collective decision-making and the wisdom of crowds. Subjective confidence is an internal estimate of the probability of being correct, and it is useful for selecting correct answers from among individual responses. In other words, confidence can compensate for, and maximize, the limits of information integration. In a visual search task, Bahrami et al. (2010) showed a “two heads are better than one” effect based on sharing of confidence between individuals to identify who was more likely to be correct. Even with no interaction between individuals, a maximum-confidence slating algorithm with higher confidence in one member of a virtual dyad showed the “two heads are better than one” effect (Koriat, 2012, 2015). Confidence has also been used to investigate the process of group decision-making. Sniezek and Henry (1989) considered frequency estimates by three-person groups to investigate whether confidence assessments are actually used in the process of the group decision-making, using estimation of 99% confidence intervals by participants. However, their results showed no evidence for the use of confidence in the group decision-making process.

It is unclear whether the findings of previous research on the wisdom of crowds can be generalized to real-life situations because most have used relatively simple deliberations and problems with single numerical estimates or multiple-choice questions. Decision-making in everyday life requires not only accurate pieces of semantic knowledge but also complex information integration where pieces of semantic knowledge should be integrated to understand the situation, to evaluate qualitatively different behavior options, and to select an optimal behavior in a goal-directed fashion. In contrast, studies of collective decision-making addressing group dynamics have often used problem-solving tasks involving consensus and team-building, which are common in real-life information integration situations. For example, managerial training and research commonly employs survival scenarios such as a desert (Lafferty, Eady and Elmers, 1974; Lafferty and Pond, 1974), a frozen area (the Winter Survival Exercise; Johnson and Johnson, 1975, 2000), and the surface of the moon, popularly known as the “NASA moon survival task” (NASA task; Hall and Watson, 1970). These tasks have common or similar procedures. Participants are placed in a hypothetical life-threatening situation and asked to rank a list of approximately 15 items in order of importance for survival. Information about the items suggests how they can be used independently or in combination with other items to meet the goals necessary for survival. Thus, these tasks can be seen as novel, complex intellective tasks with a judgmental component (Innami, 1994), an analog to the types of problems faced by managers in real life (Rogelberg, Barnes-Farrell and Lowe, 1992, p. 732). Previous studies have shown that group decisions outperform individual decisions in the Winter Survival Exercise (Miner Jr., 1984) and the NASA task (Cooke and Kernaghan, 1987; Yetton and Bottger, 1982). However, it remains unclear whether the wisdom of crowds outperforms individual decisions in similar complicated situations.

To incorporate the wisdom of crowds into survival tasks involving collective decision-making, an algorithm for aggregating many independent rank orders is needed. Recent studies have applied the wisdom of crowds to problems of rank order such as historical events (e.g., the order of US presidents) or magnitude along some physical dimension (e.g., the order of largest US cities) (Lee, Steyvers, de Young and Miller, 2012; Lee, Steyvers and Miller, 2014; Steyvers, Miller, Hemmer and Lee, 2009). The Borda count method (Marden, 2014), a technique from voting theory, is widely used to aggregate rank order data. This study adopts the Borda count method to aggregate rank orders estimated by individuals. By incorporating the aggregation of rank order data in the survival task along with weighted confidence, the study is able to compare the performance of the wisdom of crowds and collective decision-making and can further explore the process of group decision-making in a relatively realistic situation with complicated information integration.

This study employs the NASA task, which has been used extensively in decision-making literature to identify group process variables related to group performance (Burleson, Levine and Samter, 1985; Hirokawa, 1980; Innami, 1994; Linkey and Firestone, 1990; Meslec and Curşeu, 2013; Ohtsubo and Masuchi, 2004; Orpen, 1997). The NASA task asks participants to imagine a survival situation in which a spaceship crash-lands on the moon. A survivor must travel to meet the mothership, 200 miles away, and salvage 15 items from the crash, such as water, a stellar map, and a box of matches. Participants are asked to rank the 15 items in order of their relative value and utility for survival. Correct rank orders are decided by experts from the Crew Equipment Research section of the NASA manned spacecraft center in Houston, Texas, USA (Hall & Watson, 1970). Therefore, task performance can be evaluated based on an objectively correct standard. To achieve adequate rank order, participants must employ a variety of frames of reference about the 15 items and the environment of the moon’s surface. For example, to judge that the box of matches is the most useless in this situation, participants must know that oxygen is needed for combustion and that there is no oxygen on the surface of the moon. Thus, the NASA task offers an analog of multi-stage decision-making situations commonly encountered in real life.

The NASA task can assess not only the adequacy of group decisions but also synergism, resource utilization, and creativity (Hall & Watson, 1970). Decision adequacy and synergism are related to the evaluation of information integration performance; utilization of resources and creativity are related to the evaluation of the information integration process. The Decision Adequacy Index is the error score comparing individual or group rank ordering with the expert-determined order. Error scores range from 0 to 112, providing a strictly quantified index of decision adequacy for complex information integration at both individual and group levels. Based on decision adequacy, the effects of group interaction are evaluated according to the Synergism Index, namely the objective increase in group performance compared to individual performance. The Synergism Index measures both weak and strong cognitive synergies (Curşeu, Krehel, Evers and Muntean, 2014; Meslec & Curşeu, 2013; Meslec, Curşeu, Meeus and Fodor, 2014). Groups achieve weak cognitive synergy when their collective cognitive performance exceeds the average performance of individual group members and strong cognitive synergy when their collective performance exceeds the performance of the highest-performing individual in the group (Larson Jr., 2007). The Utilization of Resources Index is based on the extent to which the rank orders of individual group members are used for group decision-making. Selecting correct answers from members’ pre-discussion resources is an effective strategy in group decision-making. However, to more closely achieve the NASA experts’ judgment, it is often necessary for group members to abandon their pre-discussion resources in favor of emergent insights and solutions suggested during group interaction and information exchange. This is measured by the Creativity Index, which assesses the extent to which groups determine correct answers not present in their individual rank order resources.

This study applies these four NASA task indices (decision adequacy, synergism, resource utilization, and creativity) not only to group decision-making but also to no-weighted Borda count aggregation (standard Borda) and confidence-weighted Borda count aggregation (CW-Borda). To compute CW-Borda, the present study adopts a softmax function. The softmax function allows us to calculate not only weights of each ranking but also weights of each item within a ranking among group members by using judgments of subjective confidence. The general weighted Borda count method has used weights of each ranking across individuals (Kavallieratou and Likforman-Sulem, 2018; Miller and Steyvers, 2017). In contrast, the softmax function helps us to utilize much information regarding estimation of each rank order. Furthermore, the softmax function can modulate sensitivity of confidence weighting. Low sensitivity of the confidence weighting means that the difference in weight among group members decreases. In contrast, high sensitivity means that the weight of a higher-confident group member increases and the weight of a lower-confident group member decreases. Therefore, using group decision-making, standard Borda and CW-Borda, this study evaluates the performance and process of complicated information integration with and without forming a consensus.

Altogether, this study has six main objectives. The first objective is to confirm consistency with results of previous studies that suggested that the decision adequacy of group decision-making, standard Borda and CW-Borda outperforms the individual decision-making when complicated information integration is required. The second objective is to confirm consistency with results of previous studies that suggested that group decision-making outperforms confidence-weighted and simple aggregation by comparing the decision adequacy of group decision, standard Borda, and CW-Borda. The third objective is to confirm consistency with results of previous studies that suggested that confidence-weighted aggregation outperforms simple aggregation by comparing the decision adequacy of CW-Borda with standard Borda. The fourth objective is to compare the effects of group decision-making with standard Borda and CW-Borda in terms of the other three indices (synergism, utilization of resources, and creativity) of the NASA task. The fifth objective is to investigate the effect of confidence-weighting inherent in the group decisions from two analyses: (a) a predictability of confidence on error score using Hierarchical Linear Modeling (HLM) and (b) a utilization of confidence based on the extent to which the confidence of individual group members is used for group decision-making. Finally, the sixth objective is to use a simulation method to determine whether the effect of confidence depends on group size and sensitivity of confidence weighting. In contrast to previous studies, the study’s results showed that confidence did not improve the performance of the wisdom of crowds. Therefore, to explore a condition that improves the performance, the study used a simulation to compare CW-Borda performances with change in group size and sensitivity of confidence weighting.

## Methods

### Participants

The study participants were 119 undergraduates (100 women and 19 men; mean age = 19.4 ± 4.5 years) enrolled in a psychology class at Nara Prefectural University, Japan. Participants were randomly assigned to either a group of four persons or five persons. Of the total 25 groups in each group size condition, six were four-person groups and 19 were five-person groups. Data collection was divided into two successive academic years as a part of school course to exercise group decision-making: the first time was 10 January 2018 (55 participants) and the second time was 11 November 2018 (64 participants). Participation in the experiment was free and participants did not receive compensation. Participants provided informed consent and the study protocol was approved by the Kyoto University Ethics Committee.

## Materials

### Response sheets

The NASA task was divided into three phases: (a) individual task, (b) group task, and (c) feedback. The two phases of the individual and group decision-making were completed on each of the pages, respectively. In the phase related to the individual task, the survival situation was written at the top of the page and an answer form was presented at the bottom. The answer form included 15 rows corresponding to the 15 items and five columns including the items, rank order, usefulness, confidence, and degree of agreement with others’ answers. In the items’ column, a vertical list of the 15 items was arranged as in a previous study (Hall & Watson, 1970). Participants filled out the rank order column by ranking the 15 items in terms of their relative importance for survival. In the usefulness column, participants freely wrote how the items could be utilized in survival. In the confidence column, participants were asked to evaluate their confidence in each ranking on a 5-point scale (1 = *not at all*; 5 = *very much*). In the degree of agreement column, participants were asked to estimate how much others’ rank order would agree with their own (0% = *not at all*; 100% = *perfectly*). The phase related to the group task was similar to the individual task with the exception of the degree of agreement column, in which participants were asked to estimate the extent by which other groups’ answers would agree with their own group’s (0% = *not at all*; 100% = *perfectly*). In this paper, we did not use the responses to the questions of the degree of agreement for both individual and group phases, because of several missing values. In the phase related to the feedback, participants were provided with feedback on the results of the task. The page for the feedback contained 15 rows corresponding to 15 items and five columns: (a) individual answers, (b) group answers, (c) correct answers, (d) individual error scores, and (e) group error scores. The correct answers by NASA experts were not included in the page, and the experimenter projected the correct answers on a monitor. Participants were asked to fill in each of the columns and calculate the individual and group error scores to compare individual and group answers with the correct answers.

### Procedure

The experiment contained two main parts: individual decision-making and group decision-making. In the individual decision-making phase, the experimenter provided each participant with the response sheets and instructions on how to complete them (10 min). The participants were asked to rank the objects individually and evaluate their confidence in each ranking and its degree of agreement with others’ answers (15 min). In the group decision-making phase, five group members were predetermined randomly from a class attendance list. The experimenter projected group member lists with student codes and group positions in the classroom, and each participant was instructed to move to their assigned position (10 min). Where groups of four or five people could not be formed because of absent members, the students present were extemporarily re-grouped to form four-person or five-person groups. The experimenter then provided instructions on group decision-making. All groups were instructed to employ the method of group consensus as described by Hall and Watson (1970). First, each group member must agree upon the ranking for each of the 15 survival items before it is entered as the group decision. Second, members must avoid conflict-reducing techniques such as majority voting. Third, members must avoid readily changing their opinions simply to avoid conflict and reach agreement. To prevent intergroup influences, researchers ensured that participants interacted only within their groups, with no cross-talking between groups. After group decision-making, correct answers were projected on the monitor by the experimenter. Finally, at the end of the NASA task, each group member responded individually to questions about the group work.

### Analyses

Data analyses were conducted using the R statistical programming language (version 3.3.2). First, error scores indicating task performance, standard Borda, and CW-Borda count aggregations were calculated. Seven analyses were then performed: comparison among group decisions, standard Borda, CW-Borda, and individual decisions based on the four NASA task indices, prediction of confidence on error score and utilization of confidence for each group, and simulation of CW-Borda and standard Borda based on the change in group size and weight value by confidence.

An error score is given by the sum of the absolute differences between the ranks assigned to the items by the NASA experts and by the participants. Lower error scores indicate better performance and adequacy in terms of reasonable judgments. The individual and group rank orders were used to calculate error scores, and not the values of feedback to participants. If any group member wrote rank orders different from others, the majority rank orders of the group were used for analysis.

Standard Borda and CW-Borda were calculated for each of the 25 groups in the present study, aggregating the rank orders of four- or five-group members. In the basic Borda count method (Marden, 2014), weighted counts are assigned such that the first-choice item receives a count of *N* (where *N* is the number of items), the second-choice item receives a count of *N −* 1, and so on. These counts are summated across group members and the item with the highest count is considered the “most preferred.” To weight Borda count by confidence, the softmax function was used. The softmax function outputs a vector that represents the probability distributions of a list of potential outcomes. The softmax function is defined as follows:

1 $$ W\left({Y}_{xi}\right)=\frac{e^{k{y}_{xi}}}{\sum_{j=1}^n{e}^{ky_{xj}}}, $$

where *W*(*Y*_*xi*_) indicates a weight value for item *x* on group member *i. y*_*xi*_ is the subjective confidence (on a 5-point scale) of the ranking of item *x* for participant *i*. *k* is a sensitivity parameter of the inverse of temperature, regulating how strongly activation *y* varies with the confidence. When the *k* parameter is a lower value, the difference in the weight value among group members decreases (*k* with zero indicates no weight). Whereas, when the *k* parameter is a higher value, the weight value of a higher-confident group member increases and the weight value of a lower-confident group member decreases. The denominator is the exponential sum of all inputs for members of one group (*n* = group size). When calculating the weight value for group members on one item with a three-person group; for example, when the *k* parameter is 1, the softmax function turns confidences (logits) [1, 3, 5] into weights (probabilities) [0.02, 0.12, 0.87], which adds to 1. In the present study, the *k* parameter with 1 as a default was considered in analyses regarding the four NASA task indices, and the *k* parameter was varied from 0 to 5 in steps of 0.5 for the analyses of the utilization of confidence and simulation. Note that standard Borda is a special case of CW-Borda where *k* = 0.

Based on the above, CW-Borda count aggregation was calculated as follows. First, the softmax function calculated the weights of group members using the confidence for each item from the phase of individual task. Second, each rank order score (i.e., firstorder item = 15 points, second = 14 points, third = 13 points, etc.) was multiplied by the weights for each group member. Third, these weighted scores were summated across members, completing CW-Borda count aggregation (the item with the highest count was considered the “most preferred”).

### Comparison among group decisions, standard Borda, CW-Borda, and individual decisions based on the four NASA task indices

#### Decision adequacy

Error scores were calculated for group decisions, standard Borda, CW-Borda, and individual decisions as the difference in the correct rank order identified by experts. Average error scores of individual group members were calculated for each of groups. The one-way repeated measures analysis of variance (ANOVA) was conducted with the condition (group decisions, standard Borda, CW-Borda, and individual decisions) as the independent variable and error score as the dependent variable. We investigated whether group decision-making, standard Borda, and CW-Borda outperformed individual decision-making, and whether group decision-making outperformed standard Borda and CW-Borda.

#### Synergism

Two scores were computed for weak and strong cognitive synergy, respectively. Weak synergy was calculated as the difference between the group’s performance and the mean of the individual scores within the group; strong synergy was calculated as the difference between the group’s performance and the score of the best-performing member in the group (Larson Jr., 2007). Two separate one-way repeated measures ANOVA were then conducted, with the condition (group, standard Borda, and CW-Borda) as the independent variable and weak and strong cognitive synergy score as the dependent variable, respectively.

#### Utilization of resources

We analyzed whether group decision-making utilized more pre-discussion resources than standard Borda and CW-Borda. Specifically, the frequency with which group members’ pre-discussion decision resources were actually utilized in group decision-making was calculated. First, the number of group members whose individual decisions were the same as the group’s was counted for each item. For example, if four participants ranked an item as [1, 3, 5, 3] and the group answer was 3, the count would be 2. These counts were summated across items, and the sum was divided by product of the numbers of group members and items. This frequency rate was calculated for CW-Borda as well as group decision-making. The one-way repeated measures ANOVA was conducted to determine whether group decision-making used more pre-discussion resources than standard Borda and CW-Borda, with the condition (group, standard Borda, and CW-Borda) as the independent variable and frequency of utilization of resources as the dependent variable. Next, two regression analyses were conducted to determine whether predictors of the utilization of resources explained the error score in terms of difference between group decision-making and standard Borda and between group decision-making and CW-Borda. The independent variable was the difference in utilization of resources between group and standard Borda (or CW-Borda). The dependent variable was the difference in the error score between group and standard Borda (or CW-Borda).

#### Creativity

Creativity was assessed by calculating the frequency with which correct rankings were determined by group but were not present in group members’ pre-discussion decision resources. First, the number of group answers not present in pre-discussion resources was counted; in the group answers, the number of items with an error score within 1 was counted. For example, if three items were ranked by participant A as [3, 1, 2] and by participant B as [3, 2, 1], compared with the correct answers of [1, 2, 3], the coded numbers would be [0, 1, 1] and the final count would be 2. Then, the counts were divided by the total number of items (i.e., 15). This frequency rate was calculated for group decision-making and CW-Borda. The one-way repeated measures ANOVA was conducted to determine whether group decision-making resulted in more creative solutions than standard Borda and CW-Borda, with condition (group, standard Borda, and CW-Borda) as the independent variable and frequency of creativity as the dependent variable. Next, two regression analyses were conducted to determine whether predictors of the creativity explained the error score in terms of difference between group decision-making and standard Borda and between group decision-making and CW-Borda. The independent variable was the difference in creativity between group and standard Borda (or CW-Borda). The dependent variable was the difference in error score between group and standard Borda (or CW-Borda).

### Effects of confidence weighting inherent in the group decisions

#### Predictability of confidence on error score

HLM analysis was conducted using M-plus version 7.31 (Muthén and Muthén, 1998–2012). The predictability of confidence on error scores for each item was confirmed using two models: the random intercept model and the random intercept and slope model. The random intercept model is defined as follows:

2 $$ {\displaystyle \begin{array}{c}\mathrm{Level}\ 1:{error\ score}_{si}={\beta}_{0i}+{\beta}_1{(confidence)}_{si}+{e}_{si},\\ {}\mathrm{Level}\ 2:{\beta}_{0i}={\gamma}_{00}+{\mu}_{0i},\\ {}{\beta}_{1i}={\gamma}_{10,}\end{array}} $$

where in level 1 (within-item level), *error score*_*si*_ is the error score for subject *s* on item *i* as a dependent variable, *β*_0*i*_ is the mean error score of item *i* as an intercept, (*confidence*)_*si*_ is the subjective confidence value of subject *s* for item *i*, *β*_1_ is the slope of predictability of the confidence level, and *e*_*si*_ is the variance in error score for subject *s* around the mean of item *i* as a residual error. In level 2 (between-item level), *β*_0*i*_ is the mean error score of item *i*, *γ*_00_ is the mean error score of all items as the intercept, and *μ*_0*i*_ is the variance between the mean and the mean error score of item *i*. In the random intercept model, *β*_0*i*_ at level 1 and *γ*_00_ at level 2 have only a single value (for example, an intercept and a regression coefficient), termed *fixed effects*. In contrast, *e*_*si*_ at level 1 and *μ*_0*i*_ at level 2 have values that vary across level 1 and 2 units, respectively, termed *random effects* (i.e., a regression residual or variance).

Based on the random intercept model, a random slope was added in the random intercept and slope model, defined as follows:

3 $$ {\displaystyle \begin{array}{c}\mathrm{Level}\ 1:{error\ score}_{si}={\beta}_{0i}+{\beta}_1{(confidence)}_{si}+{e}_{si},\\ {}\mathrm{Level}\ 2:{\beta}_{0i}={\gamma}_{00}+{\mu}_{0i},\\ {}{\beta}_{1i}={\gamma}_{10}+{\mu}_{1i},\end{array}} $$

where in level 2, *β*_0*i*_ and *β*_1*i*_ are estimated with both fixed and random effects; *β*_1*i*_ is the slope value (the impact of confidence level) in an item *i*; *γ*_10_ is the mean slope across items as the intercept; and *μ*_1*i*_ is the variance of an individual’s mean slope.

#### Utilization of confidence for group decision-making

Sensitivity values of confidence weighting best fitting the group answers for each group were estimated as follows. First, error scores of CW-Borda with temperature parameter *k* from 0 to 5 by 0.5 in the softmax function were calculated for each group. Second, difference scores were calculated by subtracting error scores of group answers from that of CW-Borda for each group. The lower the difference in scores, the closer it is to the group decision. Finally, the parameter *k* was calculated to take the smallest difference value between error score of group answer and that of CW-Borda for each group.

### Simulation of CW-Borda and standard Borda count aggregations according to change in group size and sensitivity of confidence weighting

Finally, CW-Borda with standard Borda values were simulated with increasing group sizes and sensitivities of confidence weighting to investigate whether the effect of confidence depended on group size and sensitivity of confidence weighting. The simulation used data of individual rank orders and their confidence levels. Group sizes varied from 1 to 50 and sensitivities of confidence weighting varied from 0 to 5 by 0.5. In each group size, individual rank orders were randomly selected, and CW-Borda and standard Borda count aggregations were computed. This procedure was repeated 10,000 times, and a mean was computed for each group size and sensitivity of confidence weighting.

## Results

### Comparison among group decisions, standard Borda, CW-Borda, and individual decisions based on the four NASA task indices

#### Decision adequacy

**Fig. 1:**
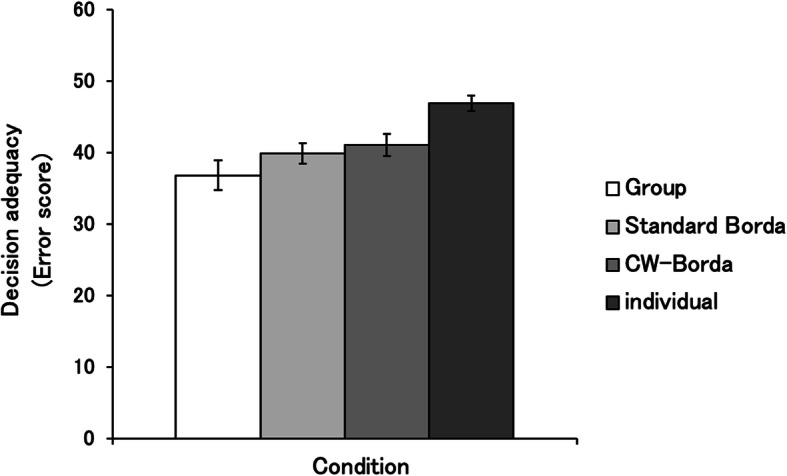
Error scores for group decisions, standard Borda, confidence-weighted (CW)-Borda count aggregations, and individual decisions. Error bars represent standard errors. Lower error scores indicate better performance

As shown in Fig. 1, ANOVA revealed a significant main effect for condition (*F* (3, 72) = 15.72, *p* < 0.001, ${\eta}_{\mathrm{P}}^2$ = 0.40). Post hoc comparisons using the Holm test indicated that error scores for group decisions (*M* = 36.88, ± 2.06), standard Borda (*M* = 39.96, ± 1.46), and CW-Borda (*M* = 41.12, ± 1.58) were significantly lower than individual decisions (*M* = 46.99, ± 1.05, *p* < 0.01). However, CW-Borda did not significantly differ from standard Borda. Contrary to the prediction, the average error score for CW-Borda was even higher than that for standard Borda.

#### Synergism

**Fig. 2:**
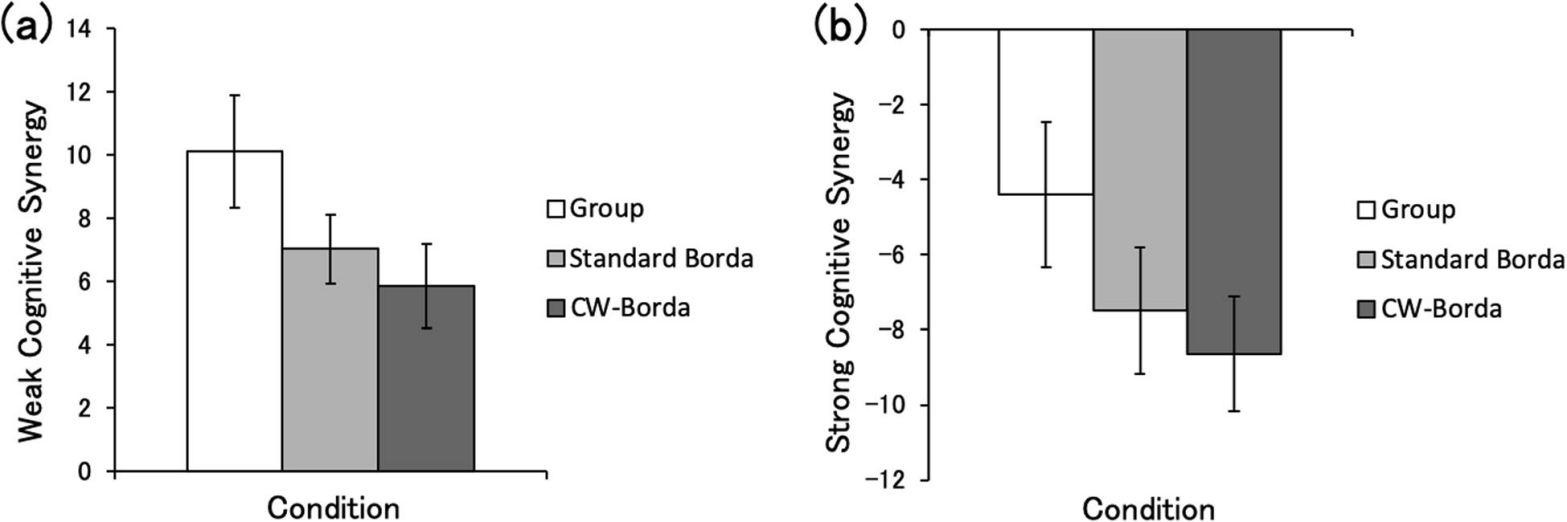
Synergy scores for group decisions, standard Borda, and confidence-weighted (CW)-Borda count aggregations. Error bars represent standard errors. **a** Weak synergy scores. Larger scores indicate performance better than the average performance of individual group members. **b** Strong synergy scores. Larger scores indicate performance better than that of the best-performing individual in the group

ANOVA for weak cognitive synergy (Fig. 2a) revealed a significant main effect for condition (*F* (2, 48) = 3.83, *p* = 0.04, ${\eta}_{\mathrm{P}}^2$ = 0.14). However, the post hoc Holm test indicated no difference among group (*M* = 10.11, ± 1.77), standard Borda (*M* = 7.03, ± 1.11), and CW-Borda (*M* = 5.87, ± 1.33). ANOVA for strong cognitive synergy (Fig. 2b) revealed a significant main effect for condition (*F* (2, 48) = 3.83, *p* = 0.04, ${\eta}_{\mathrm{P}}^2$ = 0.14). However, the post hoc Holm test indicated no difference among group (*M* = − 4.40, ± 1.93), standard Borda (*M* = − 7.48, ± 1.68), and CW-Borda (*M* = − 8.64, ± 1.53).

#### Utilization of resources and creativity

**Fig. 3:**
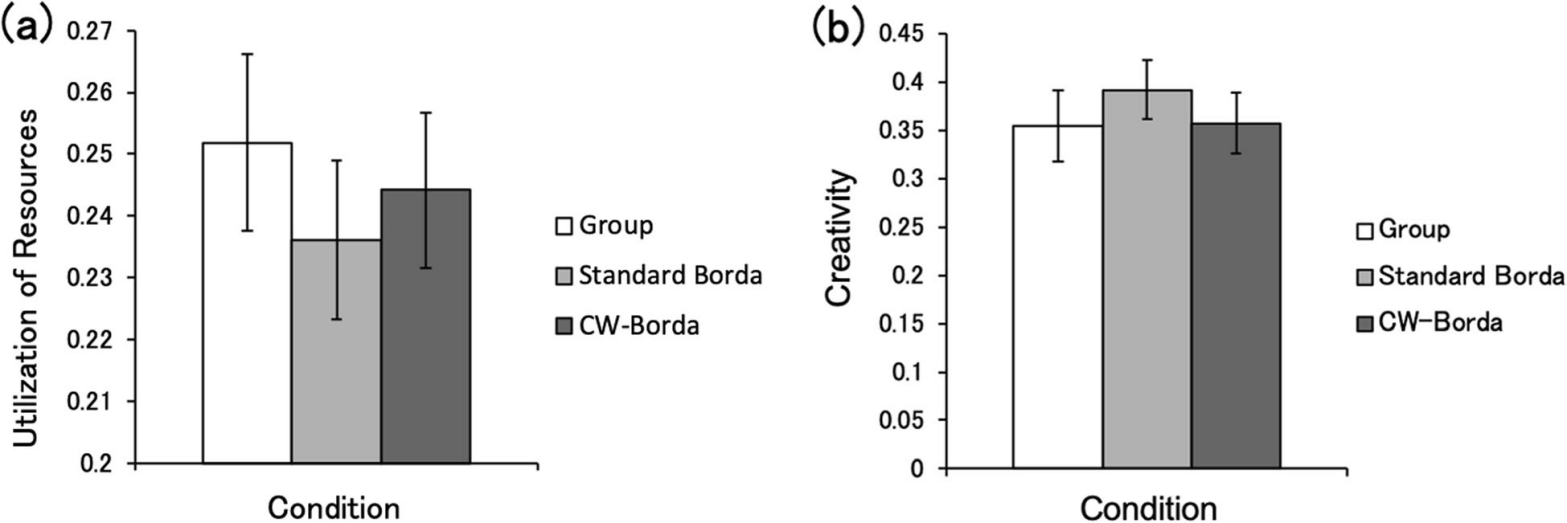
Comparison of NASA task indices between group decision-making, standard Borda, and confidence-weighted (CW)-Borda count aggregations. Error bars represent standard errors. **a** Frequency of utilization of resources. Larger values indicate more use of the rank orders of individual group members for group decision-making. **b** Frequency of creativity. Larger values indicate that groups collectively determine more correct answers that are not present in the resources of individuals’ rank orders

The other two indices, utilization of resources and creativity, relate to the process of information integration. ANOVA for utilization of resources (Fig. 3a) revealed a non-significant effect for condition (*F* (2, 48) = 0.67, *p* = 0.52, ${\eta}_{\mathrm{P}}^2$ = 0.03). Similarly, ANOVA for creativity (Fig. 3b) revealed a non-significant effect for condition (*F* (2, 48) = 0.58, *p* = 0.57, ${\eta}_{\mathrm{P}}^2$ = 0.02). These results indicate no difference in utilization of resources and creativity between collective decision-making and the wisdom of crowds in the NASA task.

**Fig. 4:**
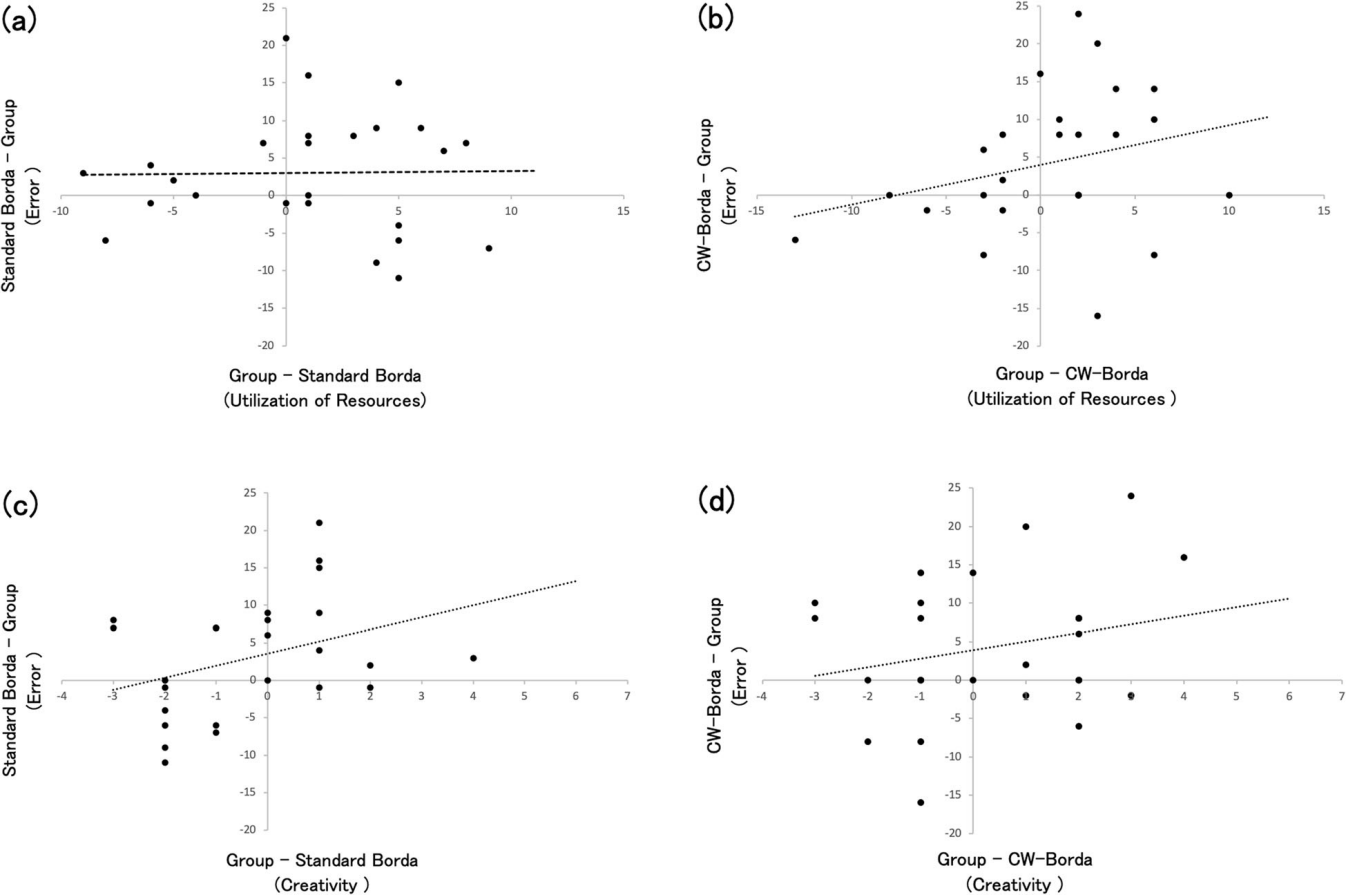
The y-axis in this figure is common to all four parts of Fig. 4 (4a, 4b, 4c, and 4d). Dotted line is least-squares best fit. **a** Relationship between utilization of resources and error score. Positive scores on the x-axis indicate that group utilizes more resources than standard Borda, while negative scores indicate that standard Borda utilizes more resources than group. Positive scores of the y-axis indicate that group performance is better than that of standard Borda, while negative scores indicate that performance of standard Borda is better than that of group. **b** Relationship between utilization of resources and error score. Confidence-weighted (CW)-Borda was used instead of standard Borda. **c** Relationship between creativity and error score. Positive scores on the x-axis indicate that group has more creativity than standard Borda, while negative scores indicate that standard Borda has more creativity than group. **d** Relationship between creativity and error score. CW-Borda was used instead of standard Borda

Next, two regression models for utilization of resources were not statistically significant: (a) the model analyzing the difference in utilization of resources between group and standard Borda predicts the difference in error score between group and standard Borda (*R*^*2*^ = 0.12, *F* (1, 23) = 3.26, *p* = 0.08) (Fig. 4a); (b) the model analyzing the difference in utilization of resources between group and CW-Borda predicts the difference in error score between group and CW-Borda (*R*^*2*^ = 0.05, *F* (1, 23) = 1.32, *p* = 0.26) (Fig. 4b). Similarly, two regression models used for creativity were not statistically significant: (a) the model analyzing the difference in creativity between group and standard Borda predicts the difference in error score between group and standard Borda (*R*^*2*^ = 0.00, *F* (1, 23) = 0.01, *p* = 0.93) (Fig. 4c); (b) the model analyzing the difference in creativity between group and CW-Borda predicts the difference in error score between group and CW-Borda (*R*^*2*^ = 0.08, *F* (1, 23) = 1.92, *p* = 0.18) (Fig. 4d). These results of regression analyses indicate no effect of the utilization of resources and creativity on performance in terms of the difference between collective decision-making and the wisdom of crowds.

### Effect of confidence weighting inherent in group decisions

#### Predictability of confidence on error score

Table 1 summarizes the results of HLM. The Akaike Information Criterion (AIC) and Bayesian Information Criterion (BIC) showed improved fitting of the random intercept and slope model compared to the random intercept model. In the random intercept and slope model, the error score was not significantly predicted by confidence (*γ*_10_: *B* = − 0.11, *p* = .43) in contrast to the random intercept model, in which error score was significantly predicted by confidence (*γ*_10_: *B* = − 0.13, *p* = .01). In the random intercept and slope model, intercept and slope variances were significant (*μ*_0*i*_: *B* = 1.27, *p* = .04; *μ*_1*i*_: *B* = 0.25, *p* = .02), indicating that the intercept and slope were different across items.

**Table 1:** Results of hierarchical linear modeling

Parameter	Random intercept model			Random intercept and slope model		
	*B*	*SE*	*p*	*B*	*SE*	*p*
Fixed effects						
*Intercept*						
*γ*_00_ (mean score)	3.52	0.36	.00**	3.63	0.34	.00**
*γ*_10_ (mean slope: confidence)	−0.13	0.05	.01*	−0.11	0.14	.43
Random effects						
*e*_*ij*_ (level 1 variance)	6.11	0.21	.01*	5.72	0.19	.00**
*μ*_0*j*_ (level 2 variance)	1.54	0.58	.01**	1.27	0.62	.04*
*μ*_1*j*_ (slope variance: confidence)				0.25	0.11	.02*
Information criteria						
AIC	8356.60			8270.98		
BIC (sample size adjusted)	8365.84			8282.48		

*AIC* Akaike Information Criterion, *BIC* Bayesian Information Criterion

^*^p < .05, ^**^p < .01

#### Utilization of confidence

**Fig. 5:**
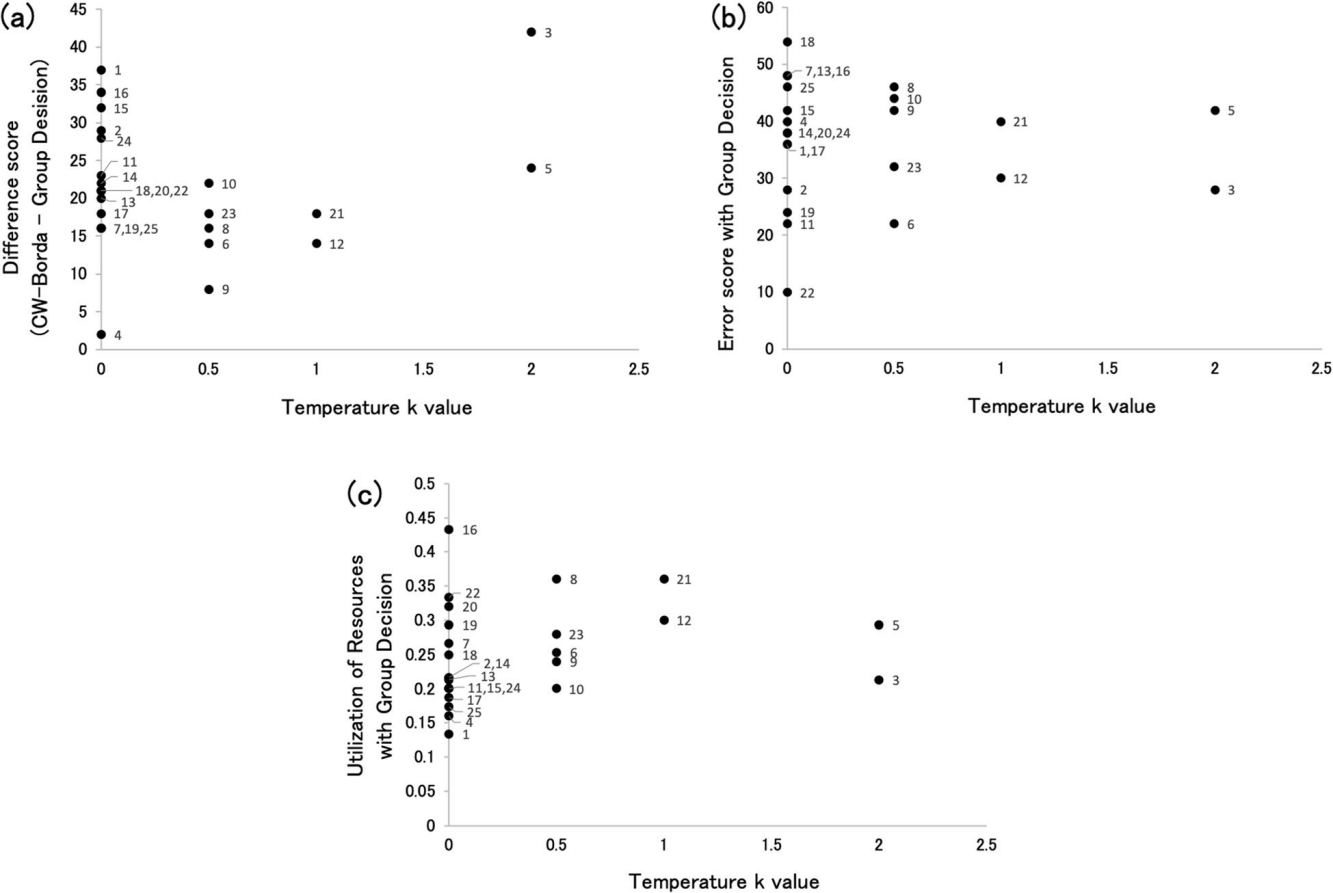
The x-axis in this figure is common to two parts of Fig. 5 (5a and 5b). The numbers on the right of the points are group IDs from 1 to 25. **a** Distribution of sensitivities of confidence weighting best matched group answers for each group. The y-axis value indicates difference scores by subtracting error scores of group answers from that of confidence-weighted (CW)-Borda. Lower error scores reflect closer to the group decision. The x-axis value indicates inverse temperature *k* values. Higher *k* values reflect that the weight value of a higher-confident group member increases and that the weight value of a lower-confident group member decreases. **b** Distribution of performances of group decisions for each group. The y-axis value indicates error scores with group decisions. Lower error scores indicate better performance. **c** Distribution of utilization of resources of group decisions for each group. The y-axis value indicates utilization of resources with group decisions. Larger values indicate more use of the rank orders of individual group members for group decision-making

As shown in Fig. 5a, the most common value was zero (64%) in optimal *k* value fitting group answers, followed by 0.5 (20%), 1 (8%), and 2 (8%). This indicates that more than half of the groups do not show the evidence of the use of confidence in their group decision. Supplemental results of error scores and utilization of resources with each group decision were shown in Fig. 5b and c.

### Simulation of CW-Borda and standard Borda count aggregations with change in group size and sensitivity of confidence weighting

**Fig. 6:**
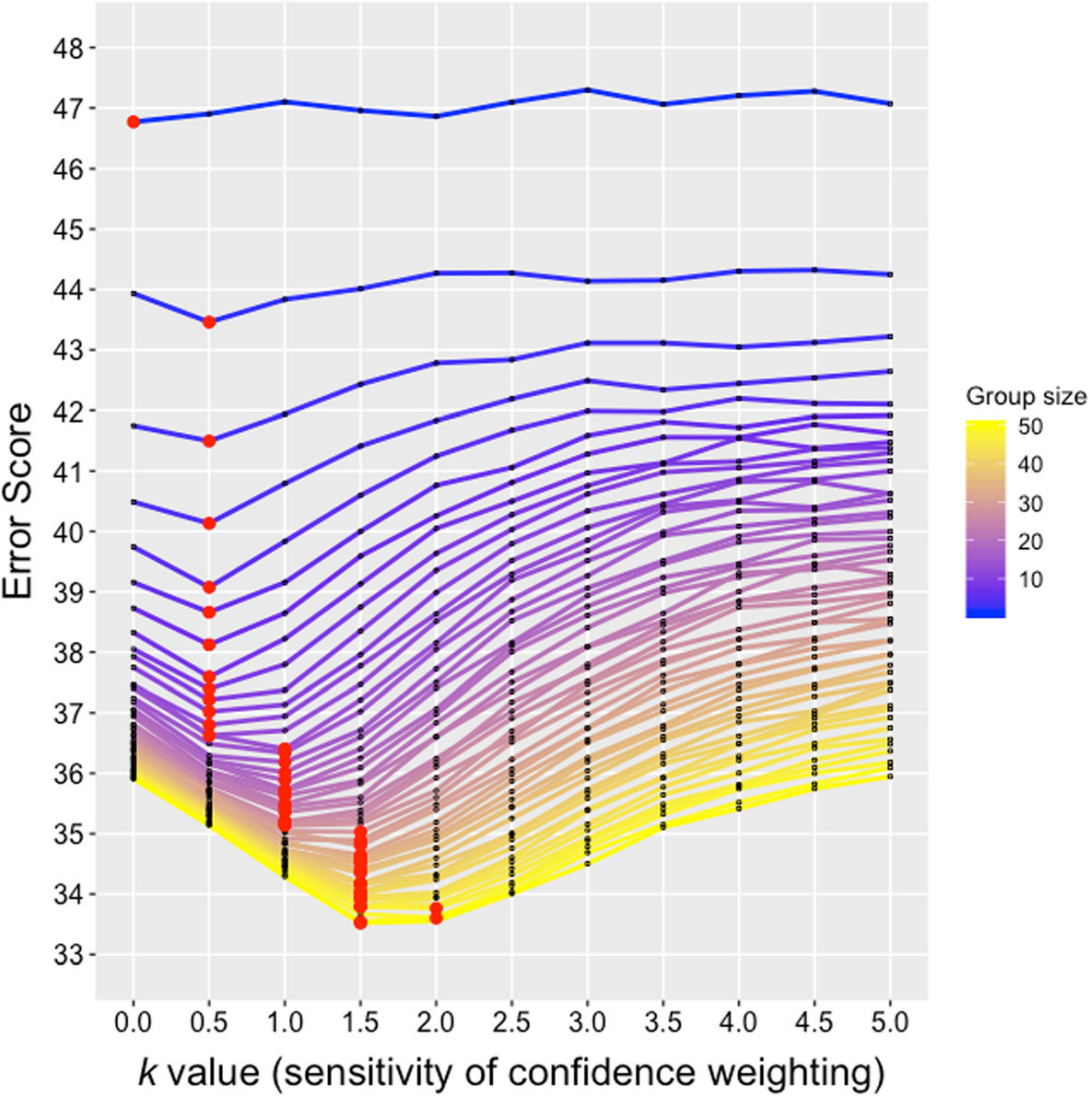
Simulation of confidence-weighted (CW)-Borda count aggregations as a function of group size and sensitivity parameter *k* of confidence weighting. Lower error scores indicate better performance. Higher *k* values indicate higher sensitivity of confidence weighting. Colors associated with lines indicate a degree of group size; yellow shows large group size and blue shows small group size. The red points indicate best *k* values of minimum error score for each group size

The results in our study showed no effect of confidence on aggregation performance, which is inconsistent with previous research. Therefore, the present study employed a simulation method to investigate whether the effect of confidence depended on group size and sensitivity of confidence weighting. The simulation analysis showed that differences in CW-Borda error scores grew larger as group size and sensitivity of confidence weighting increased. As shown in Fig. 6, the relationship between *k* value and error score showed a U shape with the best *k* values (red points) around 0 to 2. As the group size gets larger, the best *k* values and the depth of the U shape both increase. This indicates that the advantage of CW-Borda performance over the standard Borda performance (*k* value was zero) emerges by interaction between group size and sensitivity of confidence weighting. The large group size with an intermediate sensitivity (around 1 to 2) produces the best CW-Borda performance.

## Discussion

In contrast to the numerous previous studies that have employed relatively simple deliberations or problems with single numerical estimates or multiple-choice answers, this study employed the NASA task, which requires integration of realistic, complex information, to investigate the performance and process of collective decision-making and the wisdom of crowds. The results showed that groups outperformed individuals in decision adequacy, consistent with previous research (Cooke & Kernaghan, 1987; Miner Jr., 1984; Yetton & Bottger, 1982). The decision adequacy of standard Borda and CW-Borda count aggregations were also found to be superior to that of individuals, even in situations requiring complicated information integration. Comparison between group decision-making and the wisdom of crowds based on the two indices of decision adequacy and synergism indicating task performance showed no difference among group decision-making, standard Borda and CW-Borda. In terms of the utilization of resources and creativity indicating information integration process, group decision-making did not utilize more pre-discussion resources than standard Borda and CW-Borda. Similarly, group decision-making did not find more creative solutions than standard Borda and CW-Borda. The decision adequacy of CW-Borda count aggregations did not outperform that of standard Borda, inconsistent with previous research. This result suggests that weighting based on confidence may not be effective for judgments in situations requiring complicated information integration. Consistent with this suggestion, HLM analyses showed that subjective confidence had no accuracy for estimation. In addition, the analysis of utilization of confidence showed that performances in 64% of all group were closest to that of no confidence-weighted aggregation. However, the results of simulation analysis showed that CW-Borda had better adequacy than no-weighted standard Borda as group size and sensitivity of confidence weighting increased.

This study found that not only group decision-making but also the wisdom of crowds (standard Borda and CW-Borda) showed better performance than individual decision-making. However, group decision-making was not better than the wisdom of crowds, showing inconsistency with the results of Navajas et al. (2018). This inconsistency in result occurs because of no difference found in creativity and utilization of resources between group decision-making and the wisdom of crowds in complex information integration. Creativity and utilization of resources are important factors for improving decision adequacy. The Borda count algorithm is based on pre-discussion resources and limited in providing creativity. That is, the equivalence between the results for group decision-making and Borda count aggregation in creativity and utilizing resources indicate that it is difficult for groups to make optimal use of resources and present novel solutions in the NASA task. However, the decision adequacy of confidence-weighted aggregation was not superior to that of non-confidence-weighted aggregation, despite no difference in utilization of resources and creativity. This could be attributed to the characteristics of subjective confidence. Because confidence cannot accurately predict correct answers; as shown in the present study, weighting confidence would lead to worse rank aggregation. With respect to the relationships between confidence and accuracy of knowledge, current results indicated significant random variability in the confidence-accuracy relationship among different items. Consistent with this, Koriat (2015) categorized three types of items according to whether the majority agreed on the correct response (consensually correct), agreed on the wrong response (consensually wrong), or did not agree on either (non-consensual). With respect to ranking data, correlations between correct rank orders and confidence levels differed across types of questions (Lee et al., 2012), indicating that confidence was an unstable measurement for predicting answers in ranking data. Additionally, whereas judgments at the beginning and end of rank orders tend to be accurate, those in the middle tend to be less so (Lee et al., 2014; Steyvers et al., 2009). That is, ranking may include both consensually correct items at the beginning and the end of the ranking, and consensually wrong or non-consensual items in the middle. Therefore, confidence-based wisdom of crowds in ranking data may decrease the effects of confidence. However, this may be limited to relatively small groups, such as four or five people. The simulation results in this study indicated that CW-Borda tended to be more adequate than standard Borda as group size and sensitivity of confidence weighting increased, indicating that confidence is effective when group size is relatively large. Surowiecki (2004) outlined the following process to establish the wisdom of crowds. Each individual judgment contains both information and error. When there is a large enough group of diverse and independent people to make a prediction or estimate a probability, aggregating those estimates will cancel out the errors and retain the information. Therefore, the findings of the present study imply that although confidence-weighed aggregation of a small group cannot cancel out errors, and thus decreases performance, in a large group it can cancel out errors and thus increase performance. The finding thus might indicate that utilization of confidence judgment also follows one principle of the wisdom of crowds. The simulation results also indicated that fully utilizing confidence judgment in group decision-making leads to impaired performance in the situation requiring complex information integration. In this situation, partial utilization of confidence judgment could improve group decision-making, although a process of information integration in group decision-making is more complicated than wisdom of crowds.

Studies of information integration have considerable application potential in human society, which relies on groups to make important decisions. For example, the phenomenon of the wisdom of crowds has been applied not only to problems with objective answers, such as general knowledge and known values and quantities of objects, but also to open-ended problems requiring expert knowledge, such as cancer diagnosis (Kurvers et al., 2016), nuclear safety (Cooke and Goossens, 2008), and public policy (Morgan, 2014). This study’s findings suggest that the wisdom of crowds can be applied and generalized to problems of realistic, complex situations, such as multi-stage decision-making situations in which a variety of frames of reference must be employed. In such situations, maximizing utilization of resources and creativity is important to achieve reasonable expert judgments. This study indicates that the wisdom of crowds outperforms individual decision-making and is similar to collective decision-making in performance and process. Because the wisdom of crowds requires algorithms to aggregate independent judgements, it is not as flexible as collective decision-making. However, because it does not require deliberation and discussion, the wisdom of crowds has some advantages relative to collective decision-making, which requires consensus. First, the costs of aggregating individual opinions may be lower than the costs of collective decision-making, which is dependent on group interaction and information exchange. Second, the wisdom of crowds can avoid some negative biases of collective decision-making such as “groupthink” (Janis, 1982) and the “free-rider problem” (Grossman and Hart, 1980). Finally, the study’s findings suggest that weighting subjective confidence has more effect on improving performance if there is a large number of individual judgments. Thus, the study suggests the possibility that the wisdom of crowds can be expanded to problems requiring realistic, complex information integration.

This study has some limitations. First, some of the variance in error scores was not explained by the predictor of confidence at both within-item and between-item levels. This indicates that other factors may explain the remaining variance. Previous studies have shown effects of some metacognitive knowledge and heuristics other than subjective confidence. Examples include the recognition heuristic (Gigerenzer and Brighton, 2009; Goldstein and Gigerenzer, 2002), the fluency heuristic (Hertwig, Herzog, Schooler and Reimer, 2008; Jacoby and Dallas, 1981), and the familiarity heuristic (Honda, Abe, Matsuka and Yamagishi, 2011). In multiple-choice situations, these heuristics are used to infer which option has the higher value: where one of two options is recognized and the other is not (recognition heuristic); where both are recognized but one is recognized faster (fluency heuristic); and where both are recognized but one is more familiar (familiarity heuristic). In future research, it will be important to inclusively analyze the impacts of other types of metaknowledge and heuristics on decision-making performance.

Second, the generalizability of the relationship between weighting confidence and group size remains unclear. Future research must investigate whether the relationship can be generalized to relatively simple problems and answer formats (e.g., general knowledge and estimation of values or quantities of objects). This will aid in understanding the conditions in which subjective confidence elicits an effect.

Third, although this study adopted the widely used Borda count method, the best algorithm for aggregating rank orders remains unclear. Previous studies have presented a wide variety of methods for ranking aggregation, such as the average rank method (Langville and Meyer, 2012), the Condorcet method (Condorcet, 1785), the Nanson method (Nanson, 1907), Copeland’s method (Copeland, 1951), and Thurstonian models (Lee et al., 2014; Steyvers et al., 2009). A major weakness of the Borda count method is that it is easy to deliberately operate or distort the results (Langville & Meyer, 2012). Future research must investigate whether other aggregation methods compensate for the limitations of the Borda method, and which aggregation method is most appropriate for the wisdom of crowds.

## Conclusions

This study explored collective decision-making and the wisdom of crowds in a situation requiring complex information integration. The findings revealed that the wisdom of crowds performed similarly to collective decision-making, and that the effect of weighting confidence on aggregation depended on group size and sensitivity of confidence weighting. These results suggest that the wisdom of crowds is applicable to complex problem-solving tasks requiring expert knowledge. Furthermore, because the results suggest the importance of interaction between group size and sensitivity of confidence weighting in the effect of confidence-weighted aggregation, the study contributes to understanding a function of subjective confidence and expanding its potential application.

## Data Availability

The datasets used and analyzed during the current study are available from the corresponding author on reasonable request.

## Abbreviations

CW-Borda: Confidence-weighted Borda count aggregation
NASA task: NASA moon survival task
